# Cost-Effectiveness of S-Adenosyl-L-Methionine for Intrahepatic Cholestasis in the United Arab Emirates

**DOI:** 10.7759/cureus.86124

**Published:** 2025-06-16

**Authors:** Ahmad Jazzar, Kaiser Raja, Omar N Fasseeh, Ahmed Rasmy, Ahmed Gomaa, Naglaa Nasr, Ahmad N Fasseeh

**Affiliations:** 1 Gastroenterology and Hepatology, Burjeel Day Surgery Center, Abu Dhabi, ARE; 2 Gastroenterology and Hepatology, King's College Hospital London, Dubai, ARE; 3 Health Economics, Syreon Middle East, Alexandria, EGY; 4 Medicine, Alexandria University, Alexandria, EGY; 5 Hematology and Oncology, Medical Research Institute, Alexandria, EGY; 6 Medical Management, Abbott Laboratories, Dubai, ARE; 7 Market Access, Abbott Laboratories, Dubai, ARE; 8 Pharmacy, Alexandria University, Alexandria, EGY

**Keywords:** economic health evaluation, intrahepatic cholestasis, s-adenosyl methionine, united arab emirates (uae), ursodeoxycholic acid (udca)

## Abstract

Background: Intrahepatic cholestasis (IHC) is a debilitating liver condition characterized by impaired bile formation and flow, leading to the accumulation of bile acids within the liver. This condition can arise from various chronic liver diseases, including alcoholic and nonalcoholic fatty liver disease, and viral hepatitis. IHC can manifest in acute or chronic forms, with the chronic form often resulting in irreversible hepatic damage. Patients with chronic IHC experience a range of symptoms, including pruritus, jaundice, and fatigue, which significantly impact their quality of life. Current treatment options primarily include S-adenosyl-L-methionine (SAMe) and ursodeoxycholic acid (UDCA). However, their cost-effectiveness, especially in specific healthcare settings like the United Arab Emirates (UAE), has not been thoroughly investigated. This study aims to evaluate the cost-effectiveness of SAMe compared to UDCA for treating chronic IHC in the UAE, providing valuable insights for optimizing treatment protocols and allocating healthcare resources.

Materials and methods: A multi-faceted economic analysis was conducted, including cost-utility analyses (CUAs) and a cost-effectiveness analysis (CEA). The primary CUA compared SAMe to UDCA, focusing on their impact on pruritus and fatigue. The CEA compared SAMe and UDCA based on changes in key biochemical parameters. An additional CUA compared both SAMe and UDCA to no treatment, considering a broader range of IHC-related comorbidities (jaundice, fatigue, pruritus, and depressed mood).

Results: In the base case CUA, SAMe had an incremental cost-utility ratio (ICUR) of 44,448 AED per quality-adjusted life year (QALY) compared to UDCA. In the CEA, SAMe demonstrated cost-effectiveness in reducing alkaline phosphatase but was less cost-effective for other biomarkers compared to UDCA. In the broader CUA, SAMe had a lower ICUR (41,202 AED/QALY) than UDCA (70,090 AED/QALY) when compared to no treatment. Sensitivity analyses confirmed the robustness of these findings.

Conclusions: SAMe demonstrates cost-effectiveness compared to UDCA and no treatment for chronic IHC in the UAE, particularly when considering its broader impact on comorbidities. These findings support the integration of SAMe into treatment protocols for IHC, potentially improving patient outcomes and optimizing healthcare resource allocation. Further research is needed to address data gaps, especially regarding UDCA's effects on jaundice and depressed mood in IHC.

## Introduction

Despite advancements in liver disease treatments, intrahepatic cholestasis (IHC) remains an unmet medical challenge, leaving patients struggling with debilitating symptoms that severely impact their quality of life.

IHC is a liver condition characterized by impaired bile formation and flow, leading to the accumulation of bile acids within the liver. This condition is commonly linked to various chronic liver diseases, including alcoholic liver disease, nonalcoholic fatty liver disease, and viral hepatitis. It is essential to note that IHC can present in either acute or chronic forms; however, the focus of this analysis is specifically on the chronic form of IHC [1]. The symptoms of IHC, including pruritus, jaundice, and fatigue, severely affect patients' quality of life and overall health outcomes [2].

Specific prevalence data for IHC in the United Arab Emirates (UAE) is limited. However, it is anticipated that a substantial number of adults with chronic liver diseases in the region are affected by this condition [3].

The primary treatment approach focuses on addressing the underlying etiology, which is more feasible in acute IHC but presents a significant challenge in chronic cases where irreversible hepatic damage has occurred. Consequently, symptom management assumes a critical role in enhancing the quality of life for patients with chronic IHC. Therapeutic interventions include ursodeoxycholic acid (UDCA), S-adenosyl-L-methionine (SAMe), and cholestyramine [1,4].

In the context of chronic IHC, where symptom management is paramount, SAMe, a natural derivative of the amino acid methionine, has emerged as a promising treatment option for IHC. Research indicates that SAMe not only improves biochemical parameters related to liver function but also effectively alleviates symptoms such as pruritus, fatigue, depression, and jaundice. Its ability to address both clinical manifestations and underlying liver damage makes SAMe a potentially more comprehensive treatment for IHC compared to existing options. These advantages make SAMe a critical candidate for further evaluation of its cost-effectiveness and adoption in the UAE healthcare system, which could lead to more informed decision-making regarding resource allocation and treatment strategies [4-6].

The primary objective of this study is to evaluate the cost-utility of SAMe compared to UDCA in adult patients with chronic IHC within the UAE. This evaluation will involve a comprehensive economic analysis that includes a cost-effectiveness analysis (CEA), focusing on physiological parameters, and a cost-utility analysis (CUA), assessing the incremental cost-utility ratio (ICUR) expressed in terms of quality-adjusted life years (QALYs) gained.

This article was previously presented as an abstract at the 2025 DUPHAT conference.

## Materials and methods

To evaluate the cost-effectiveness of SAMe for treating IHC in the UAE, we employed a multifaceted approach encompassing three distinct scenarios.

Our primary analysis (base case) centered on a CUA, directly comparing SAMe to UDCA. This analysis focused specifically on the impact of each treatment on two predominant and quality-of-life-impacting symptoms of IHC: pruritus and fatigue. We prioritized these symptoms for the CUA due to the availability of data on SAMe's impact in these areas and their significant impact on patients' well-being, allowing for a meaningful assessment of health utility improvements associated with SAMe treatment. While other symptoms are relevant to IHC, comprehensive data on the relative efficacy of SAMe and UDCA across all symptoms were not available.

In a second scenario, we conducted a CEA to compare SAMe and UDCA based on their effects on key biochemical parameters. This analysis encompassed changes in alanine aminotransferase (ALT), aspartate aminotransferase (AST), alkaline phosphatases (ALP), gamma-glutamyl transpeptidase (GGT), conjugated bilirubin, and total bilirubin levels. This approach provided a comprehensive evaluation of the direct biochemical impact of SAMe treatment relative to UDCA, focusing on objective markers of liver function.

Finally, to address the limitations posed by data scarcity regarding UDCA, particularly its impact on a broader range of IHC-related comorbidities, we performed a third scenario involving separate CUAs. In this scenario, SAMe was compared to no treatment, and UDCA was also compared to a no-treatment control. These analyses incorporated a wider set of comorbidities associated with IHC, including jaundice, fatigue, pruritus, and depressed mood. This indirect comparison allowed us to capture the full potential health utility gains of SAMe by utilizing available data on its impact on these comorbidities in the absence of treatment, which would not be fully captured in the direct comparison with UDCA due to limited data availability for the latter. Therefore, this scenario was crucial for capturing the full potential of SAMe and estimating its full benefits.

Model design

**Figure 1:**
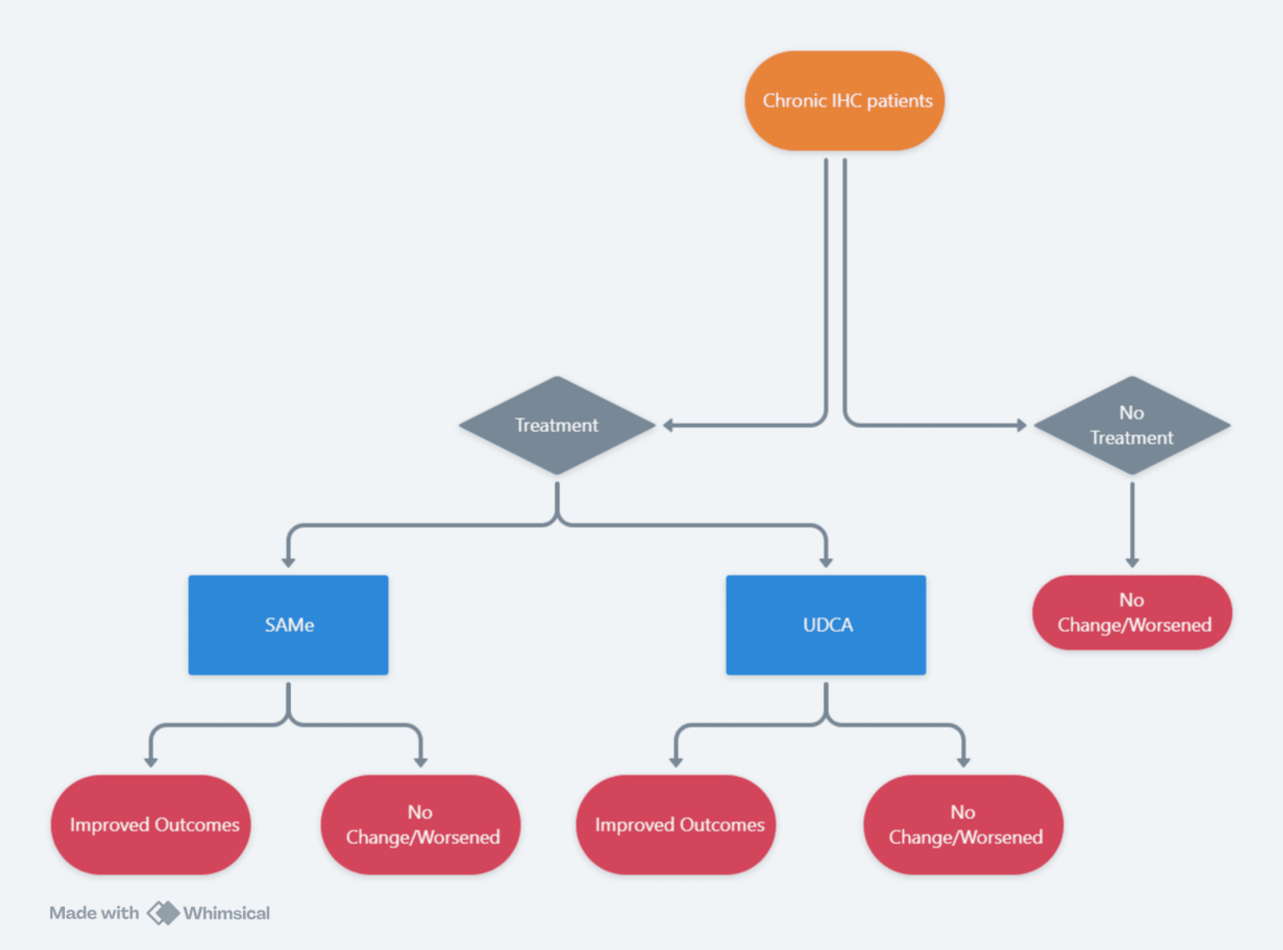
Model design IHC: intrahepatic cholestasis, SAMe: S-adenosyl-L-methionine, UDCA: ursodeoxycholic acid

A decision-analytic model was developed to evaluate the cost-effectiveness of SAMe for treating chronic IHC in adult patients in the UAE. The model employed a multi-scenario approach, as shown in Figure 1, incorporating both a CEA and CUAs, each with different time horizons tailored to the specific outcomes being measured. The analyses were conducted from the perspective of private payers (insurance) in the UAE, considering only direct medical costs.

The CEA scenario employed a two-week time horizon to capture changes in physiological parameters, as biomarker changes typically occur earlier than symptomatic improvements. In contrast, the CUAs used a six-month time horizon to adequately capture improvements in symptoms and quality of life, aligning with the timeline observed in the literature for these outcomes. Discounting was not applied, given the relatively short time horizon of the analyses.

The study focuses on adult patients diagnosed with chronic IHC in the UAE. The primary intervention is SAMe, which has shown promise in improving symptoms and biochemical parameters in patients with IHC. Data from the literature suggest that SAMe improves several biochemical liver parameters, including ALT, AST, ALP, GGT, total bilirubin, and conjugated bilirubin. Additionally, it reduces fatigue, symptoms of depressed mood, pruritus, and jaundice, which are common manifestations of IHC. By mitigating oxidative stress and inflammation, SAMe has the potential to improve the physiological and psychological burdens associated with IHC.

In this health technology assessment, UDCA was selected as the primary comparator for SAMe. This choice aligns with the UAE HTA guidelines, which stipulate that the comparator should be an authorized and commonly used health technology in the investigated indication. UDCA meets these criteria as it is approved and commonly used for the treatment of IHC in the UAE, supported by scientific evidence and validated in current clinical practice guidelines. UDCA's mechanism of action, which involves modifying bile acid composition to reduce bile acid toxicity, addresses the underlying pathology of IHC. Furthermore, both SAMe and UDCA are indicated for the same patient population and target similar symptoms of IHC, ensuring a relevant comparison. Additionally, according to the guidelines, when no effective therapy is available or reimbursed, a no-treatment arm can be added. Since both are currently used for the treatment of IHC, a no-treatment arm was still added to quantify the full potential and benefits of SAMe.

Data sources and model inputs

Data sources for model inputs included baseline comorbidity data, drug efficacy data, cost data, utility values, and laboratory test results. The clinical efficacy data for SAMe and UDCA were gathered from available literature assessing their impact on liver enzymes (e.g., ALT, AST, ALP, GGT) and symptoms (e.g., pruritus, fatigue, jaundice). Cost data were derived from UAE healthcare sources, using market prices of SAMe and UDCA. Utilities, represented as QALYs, were also incorporated to assess the overall impact on patient quality of life. These inputs were integral to calculating the incremental cost-effectiveness ratios (ICERs), which provide a comparative assessment of the value offered by each intervention. The model inputs, along with their sources, are detailed in Table 1.

**Table 1:** Model inputs and sources SAMe: S-adenosyl-L-methionine, UDCA: ursodeoxycholic acid, IHC: intrahepatic cholestasis, ALP: alkaline phosphatases, AST: aspartate aminotransferase, ALT: alanine aminotransferase, GGT: gamma-glutamyl transpeptidase, AED: United Arab Emirates Dirham, MOH: Ministry of Health, UAE: United Arab Emirates

Baseline comorbidities data									
Comorbidity	Percentage of patients with comorbidity		Source						
Fatigue	81.0%		Perlamutrov et al. 2014 [5]						
Jaundice	20.0%		Perlamutrov et al. 2014 [5]						
Pruritus	48.8%		Manzillo et al. 1992 [6]						
Depressed mood	87.6%		Perlamutrov et al. 2014 [5]						
Drugs’ efficacy									
SAMe									
Reduction in the proportion of patients with fatigue	64%		Adapted from Perlamutrov et al. 2014 [5]						
Reduction in the proportion of patients with jaundice	86%		Adapted from Perlamutrov et al. 2014 [5]						
Reduction in the proportion of patients with pruritus	86%		Adapted from Manzillo et al. 1992 [6]						
Reduction in the proportion of patients with depressed mood	43%		Adapted from Perlamutrov et al. 2014 [5]						
UDCA									
Reduction in the proportion of patients with Pruritus	67%		Adapted from Manzillo et al. 1992 [6]						
Reduction in the proportion of patients with fatigue	9%		Adapted and digitized from Heathcote 1994 [7]						
Costs									
Drug	Cost/pack		Number of tablets/packs		Dose per tablet in mg	Daily dose in mg		Source	
SAMe	167.0 AED		20		500	1,000		MOH published prices in UAE (July 2, 2024)	
UDCA	169.54 AED		50		500	1,000		MOH published prices in UAE (July 2, 2024)	
Utility data									
Comorbidity	Value	Source	Comment						
Baseline utility of IHC patients	0.79	Papatheodoridi et al. 2023 [8]							
Fatigue	0.79*	Adapted from Younossi et al. 2020 [9]	*Multiplicative disutility						
Jaundice	0.94*	Adapted from McPhail et al. 2021 [10]							
Pruritus	0.87*	Adapted from Younossi et al. 2020 [9]							
Depressed mood	0.88*	Adapted from Kim et al. 2018 [11]							
Laboratory test values									
Lab test	Baseline lab values	Baseline source	Percentage change with SAMe*	Source			Percentage change with UDCA*		Source
ALP (U/L)	289.20	Manzillo et al. 1992 [6]	-20%	Adapted from Manzillo et al. 1992 [6]			+12%		Adapted from Kondrackiene et al. 2005 [12]
AST (U/L)	82.80	Manzillo et al. 1992 [6]	-32%	Adapted from Manzillo et al. 1992 [6]			-61%		Adapted from Kondrackiene et al. 2005 [12]
ALT (U/L)	74.40	Manzillo et al. 1992 [6]	-36%	Adapted from Manzillo et al. 1992 [6]			-60%		Adapted from Kondrackiene et al. 2005 [12]
GGT (U/L)	114.00	Manzillo et al. 1992 [6]	-38%	Adapted from Manzillo et al. 1992 [6]			-8%		Adapted from Kondrackiene et al. 2005 [12]
Conjugated bilirubin (mg/dl)	0.99	Manzillo et al. 1992 [6]	-48%	Adapted from Manzillo et al. 1992 [6]			NA		NA
Total bilirubin (mg/dl)	2.59	Manzillo et al. 1992 [6]	-45%	Adapted from Manzillo et al. 1992 [6]			-23%		Adapted from Kondrackiene et al. 2005 [12]

Model calculations

Utility Calculations

This study estimated utility values for patients with IHC at both baseline and post-treatment, considering the impact of four key comorbidities: fatigue, jaundice, pruritus, and depressed mood. The calculations were performed in two main parts: determining the baseline utility and the utility after treatment for each treatment arm.

Determining the proportion of patients with comorbidities after treatment: To accurately assess post-treatment utility, the proportion of patients experiencing each comorbidity after treatment was first calculated. This was achieved using the following formula:

\[ \text{After treatment % of patients with comorbidity} = \left( \text{Baseline % of patients with comorbidity} \times \text{% Reduction in proportion of patients with comorbidity with intervention} \right) \]

This means that for each comorbidity, the baseline percentage of patients experiencing the condition was adjusted by the percentage reduction in that comorbidity observed after treatment. This calculation yields the updated proportion of patients experiencing each comorbidity after the intervention in each treatment arm.

Baseline utility calculation: The baseline utility for each treatment arm was calculated using a multiplicative approach. This involved combining the baseline quality of life of IHC patients with the weighted utility decrements associated with each comorbidity. The calculation is described as follows:

For each comorbidity (fatigue, jaundice, pruritus, and depressed mood): For Step 1 (weighted utility decrement), multiply the baseline percentage of patients experiencing the comorbidity by the multiplicative utility decrement associated with that comorbidity. For Step 2 (weighted utility value), multiply the percentage of patients not experiencing the comorbidity (calculated as 1 minus the baseline percentage * 1) by the baseline quality of life of IHC patients (the utility value in the absence of any comorbidities). For Step 3 (combined comorbidity impact), add the results from Step 1 and Step 2. This represents the combined impact of that comorbidity on overall utility, accounting for both patients with and without the comorbidity.

The overall baseline utility was determined by multiplying the baseline quality of life of IHC patients by the combined comorbidity impacts calculated in Step 3 for each of the four comorbidities.

Below is the equation for baseline utility:

\begin{align*} \text{Baseline Utility} = \Bigg[ &\left( \text{Baseline % of patients with Fatigue, Pruritus, Jaundice and depressed mood} \right. \\ &\quad \times \text{Multiplicative Utility Decrement for Fatigue, Pruritus, Jaundice and depressed mood} \\ &+ \left( 1 - \text{Baseline % of patients with Fatigue, Pruritus, Jaundice and depressed mood} \times 1 \right) \Bigg] \\ &\quad \times \text{Baseline utility of IHC} \end{align*}

Post-treatment utility calculation: The post-treatment utility for each treatment arm was calculated using the same multiplicative approach as the baseline utility. However, instead of using the baseline percentages of patients with each comorbidity, the after-treatment percentages (calculated in Section 1) were used. This accounts for the changes in comorbidity prevalence following each intervention.

Below is the equation for after-treatment utility:

\[ \begin{aligned} \text{After Treatment Utility} = \Big[ & \left( \text{After treatment % of patients with Fatigue, Pruritus, Jaundice, and depressed mood} \right. \\ & \quad \times \text{Multiplicative Utility Decrement for Fatigue, Pruritus, Jaundice, and depressed mood} \\ & + \left( 1 - \text{After treatment % of patients with Fatigue, Pruritus, Jaundice, and depressed mood} \times 1 \right) \Big] \\ & \quad \times \text{Baseline utility of IHC} \end{aligned} \]

Cost-effectiveness calculations

The CEA evaluated the clinical impact of SAMe and UDCA in treating IHC by comparing changes in key biochemical markers over a two-week period. Data on baseline and post-treatment laboratory values for ALT, AST, ALP, GGT, conjugated bilirubin, and total bilirubin were collected from the published literature. These markers are routinely monitored in IHC patients and serve as indicators of treatment efficacy.

Methodology

The primary aim of the analysis was to determine the cost per unit reduction for each biochemical marker. This metric provides insight into the relative cost-effectiveness of each intervention by quantifying the cost required to achieve a specific unit change in each marker. To achieve this, the following steps were undertaken:

Incremental change in laboratory values: The change from baseline in each laboratory parameter (ALT, AST, ALP, GGT, conjugated bilirubin, and total bilirubin) was calculated for both SAMe and UDCA after two weeks of treatment. The incremental change was then determined by calculating the difference between the changes observed with SAMe and UDCA.

For the incremental cost, the difference in treatment costs between SAMe and UDCA over the two-week period was calculated. On the other hand, the cost per unit reduction for each laboratory parameter was calculated by dividing the incremental cost (from Step 2) by the incremental change in the laboratory value (from Step 1). The cost per unit reduction was calculated using the following formula:

\( {Cost\ per\ unit\ reduction} = \frac{ \left( \text{Baseline lab value} \times \text{% Change in lab value with intervention} \right) - \left( \text{Baseline lab value} \times \text{Reduction in lab value with comparator} \right) }{ \text{Cost of intervention} - \text{Cost of comparator} } \)

The resulting cost per unit reduction provides a standardized measure for comparing the cost-effectiveness of SAMe and UDCA across different biochemical markers. A reduction in the cost per unit indicates greater cost-effectiveness, suggesting that a particular treatment achieves a larger improvement in the marker for a given cost.

Cost calculations

The total cost of each intervention in the model was estimated by combining two primary components: the direct cost of the drug itself and the costs associated with managing comorbidities. These costs were calculated over the specified time horizon of the model.

Direct Drug Costs

The direct cost of each intervention (SAMe, UDCA, or No Treatment) was calculated as follows:

${Direct\ Drug\ Cost} = \left( \text{Monthly Drug Cost} \times \text{Time Horizon in months} \right)$

This calculation provides the cumulative cost of the drug over the chosen duration of the model.

Comorbidity Management Costs

The model incorporates the costs of managing comorbidities associated with IHC, including fatigue, jaundice, pruritus, and depressed mood. However, due to a lack of data on the specific costs of managing these comorbidities in the UAE context, these costs were set to zero in the current model. While this represents a limitation of the current analysis, it provides a conservative estimate of the cost-effectiveness of the interventions. The comorbidity management cost for each intervention is calculated as follows:

For each comorbidity, multiply the proportion of patients experiencing the comorbidity by the cost of managing that comorbidity.

${Comorbidity\ Costs} = \left( \text{Proportion of patients with comorbidity} \times \text{Costs of managing comorbidity} \right) \times \text{Time Horizon}$

Sum the costs calculated in the previous step for all four comorbidities to get the total comorbidity management cost for a single patient. Lastly, multiply the total comorbidity management cost per patient by the time horizon (in months) to obtain the cumulative cost of managing comorbidities over the model's duration.

Total Intervention Cost

The total intervention cost is the sum of the direct drug cost and the costs associated with comorbidity management.

${Total\ Cost} = \left( \text{Drug Cost} + \text{Comorbidities Cost} \right)$

Handling data scarcity in model development

Data scarcity, particularly in head-to-head comparisons of different interventions, presented a significant challenge in developing this model. While published data were available on the effectiveness of SAMe in reducing the proportion of patients experiencing all relevant comorbidities (fatigue, jaundice, pruritus, and depressed mood), similar comprehensive data for UDCA were lacking. Specifically, data on UDCA's impact were available only for fatigue and pruritus, but not for jaundice or depressed mood.

This data gap posed a challenge for conducting a direct CUA between SAMe and UDCA. The absence of evidence regarding UDCA's effect on jaundice and depressed mood does not necessarily imply a lack of effect but rather reflects a limitation in the available research. To ensure a fair and balanced comparison given these limitations, a conservative approach was adopted for the CUA.

Methodological Approach for Direct CUA (SAMe vs. UDCA)

In the direct CUA comparing SAMe and UDCA, the after-treatment utility calculation considered only those comorbidities for which data were available for both drugs. Consequently, only the effects on fatigue and pruritus were incorporated into this specific analysis.

Implications of the Approach

While this approach provides a fair comparison based on available data, it is important to acknowledge that it may underestimate the overall effectiveness of SAMe. By excluding jaundice and depressed mood from the comparison, the potential benefits of SAMe in these areas are not captured. However, this approach was deemed more appropriate than assuming that UDCA does not affect jaundice or depressed mood simply due to a lack of data.

Addressing Data Limitations in Other Analyses

It is essential to note that this limitation primarily affected the direct comparison of CUA between SAMe and UDCA. In the broader analyses comparing each intervention to no treatment, a more comprehensive assessment of SAMe's impact on all four comorbidities (fatigue, jaundice, pruritus, and depressed mood) was possible, as data were available for these outcomes in the SAMe and no-treatment groups.

Sensitivity analysis

Both deterministic sensitivity analysis (DSA) and probabilistic sensitivity analysis (PSA) were conducted to evaluate the model's robustness. DSA was conducted by systematically varying the key-input parameters by ±10%. At the same time, a PSA was conducted by performing 1,000 iterations to account for uncertainty in model inputs, providing distributions of costs and QALYs for each intervention. The distributions assumed for the different categories of inputs are shown in Table 2.

**Table 2:** Statistical distribution of the variable types included in the PSA * Base case values and standard errors for all variables are detailed in the appendix. Standard errors were considered to be 10% of the mean value. PSA: probabilistic sensitivity analysis

Parmeter	Distribution
Utilities*	Beta
Utility decrements*	Beta
Baseline lab values*	Log normal
Treatment effects (reduction in biomarker values)*	Log normal
Costs*	Gamma

Cost-effectiveness threshold

The cost-effectiveness threshold was based on UAE economic standards, specifically using a threshold value of 161,854 AED per QALY gained, which was calculated using an economic evaluation tool that considered the country’s GDP per capita and the incremental benefits of the interventions [13].

## Results

Base case: cost-utility analysis of S-adenosyl-L-methionine and ursodeoxycholic acid considering pruritus and fatigue only

In this scenario, a CUA of SAMe vs. UDCA treatment was conducted, considering only their impact on pruritus and fatigue. The results indicate that SAMe incurs an additional cost of 1,786 AED while providing an incremental benefit of 0.040 QALYs, as shown in Table 3. Thus, the ICUR is 44,448 AED/QALY, reflecting that SAMe is cost-effective (based on the 161,854 AED cost-effectiveness threshold, as explained in the Materials and Methods section).

**Table 3:** Results of base case scenario (Scenario 2) QALY: quality-adjusted life years, ICUR: incremental cost-utility ratio, AED: United Arab Emirates Dirham, SAMe: S-adenosyl-L-methionine, UDCA: ursodeoxycholic acid

Parameter	SAMe	UDCA	Incremental values
Cost (AED)	3,006	1,220	1,786
QALYs	0.365	0.325	0.04
ICUR (cost/QALY)	44,448 AED/QALY		

Cost-effectiveness analysis of S-adenosyl-L-methionine and ursodeoxycholic acid (chemical biomarkers only)

In this scenario, we conducted CEA by comparing SAMe to UDCA with respect to the reduction in biochemical markers, and we used the cost of treatment over two weeks to calculate the cost-effectiveness.

Variations in the effects of SAMe and UDCA were observed for various biomarkers (Table 4). SAMe showed reductions in all markers, including ALP (57.6 U/L), AST (26.4 U/L), ALT (27.0 U/L), GGT (43.2 U/L), conjugated bilirubin (0.5 mg/dL), and total bilirubin (1.2 mg/dL). UDCA, on the other hand, demonstrated reductions in AST (50.5 U/L), ALT (44.4 U/L), GGT (9.6 U/L), and total bilirubin (0.6 mg/dL), while ALP increased by 35.6 U/L. Conjugated bilirubin data for UDCA were not available. UDCA was more effective than SAMe in reducing AST and ALT levels, whereas SAMe showed greater reduction in GGT, total bilirubin, and ALP.

**Table 4:** Results of the CEA of SAMe and UDCA (chemical biomarkers only) * Negative sign means an increase from baseline, ** UDCA was dominant in ALT and AST SAMe: S-adenosyl-L-methionine, UDCA: ursodeoxycholic acid, ICER: incremental cost-effectiveness ratio, AED: United Arab Emirates Dirham, ALP: alkaline phosphatases, AST: aspartate aminotransferase, ALT: alanine aminotransferase, GGT: gamma-glutamyl transpeptidase, CEA: cost-effectiveness analysis

Intervention	Cost of treatment/2 weeks	ALP (U/L)	AST (U/L)	ALT (U/L)	GGT (U/L)	Conjugated bilirubin (mg/dl)	Total bilirubin (mg/dl)
SAMe (change in biomarkers)	AED 251	57.6	26.4	27.0	43.2	0.5	1.2
UDCA (change in biomarkers)	AED 102	-35.6*	50.5	44.4	9.6	NA	0.6
Delta change in physiological parameter (SAMe-UDCA)	-	93.2	-24.1	-17.4	33.6	NA	0.6
SAMe vs. UDCA cost/unit reduction (ICER)	-	1.6 AED	-6.2** AED	-8.5** AED	4.4 AED	NA	261.7

The cost of unit reduction of each biomarker between SAMe and UDCA, that is, the ICER, is shown in Table 4. The lowest ICER was for reduction in ALP (1.6 AED/unit reduction), whereas the highest ICER was for reduction in total bilirubin (261.7 AED/unit reduction).

Cost-utility analyses of S-adenosyl-L-methionine vs. no treatment and ursodeoxycholic acid vs. no treatment

In this scenario, SAMe and UDCA were indirectly compared by using a common comparator (no treatment) as detailed in Table 5.

**Table 5:** Incremental CUA of interventions against no treatment QALY: quality-adjusted life year, ICUR: incremental cost-utility ratio, AED: United Arab Emirates Dirham, SAMe: S-adenosyl-L-methionine, UDCA: ursodeoxycholic acid, CUA: cost-utility analysis

Comparison	SAMe vs. no treatment	UDCA vs. no treatment
Incremental costs	3,006 AED	1,220 AED
Incremental QALYs	0.073 QALYs	0.017 QALYs
ICUR (cost/QALY)	41,202 AED/QALY	70,090 AED/QALY

**Figure 2:**
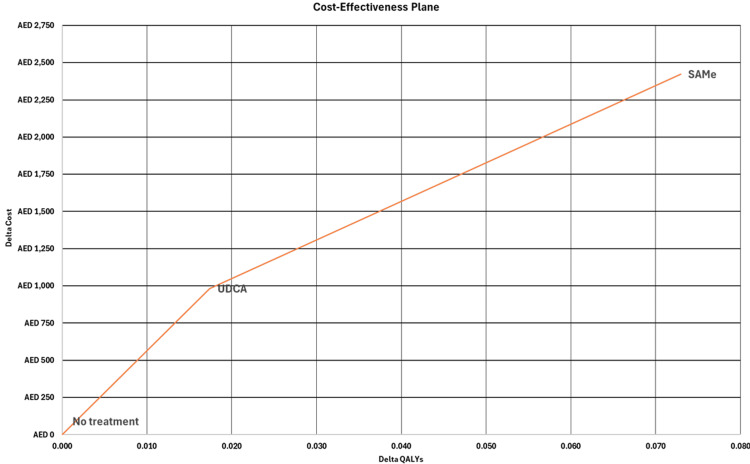
Cost-effectiveness plane for indirect comparison between SAMe and UDCA SAMe: S-adenosyl-L-methionine, UDCA: ursodeoxycholic acid, AED: United Arab Emirates Dirham

SAMe, compared to no treatment, had an incremental cost of 3,006 AED and yielded an incremental 0.073 QALYs, resulting in an ICUR of 41,202 AED/QALY. However, UDCA, compared to no treatment, had an incremental cost of 1,220 AED, but only yielded 0.017 additional QALYs, leading to a higher ICUR of 70,090 AED/QALY. We demonstrate the results of the indirect comparison of SAMe and UDCA on the cost-effectiveness plane, as shown in Figure 2.

Results of the deterministic sensitivity analysis

DSA for SAMe vs. UDCA

**Figure 3:**
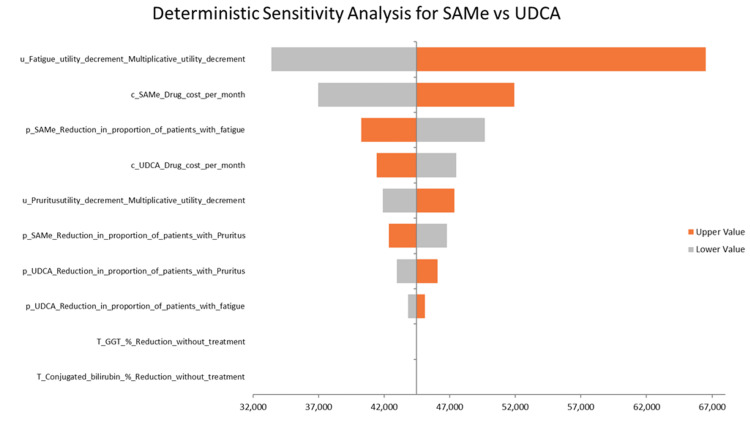
DSA of SAMe vs. UDCA (base case) DSA: deterministic sensitivity analysis, SAMe: S-adenosyl-L-methionine, UDCA: ursodeoxycholic acid

The base-case ICUR for SAMe vs. UDCA is 44,448 AED/QALY, which is 115,552 AED below the willingness-to-pay threshold of 161,854 AED, indicating a favorable cost-effectiveness profile. The most influential parameter is the disutility value of fatigue, as shown in Figure 3, with the ICUR ranging from 33,377 AED to 66,508 AED, corresponding to a -24.89% to +49.64% change from the base-case ICUR. The second most influential parameter is the monthly cost of SAMe, where the ICUR varies between 36,966 AED and 51,931 AED, reflecting a change of -16.81% to +16.80%. Lastly, the efficacy of SAMe in reducing fatigue shows an ICUR range of 40,230 AED to 49,654 AED, translating to a -9.48% to +11.71% variation. Despite these sensitivities, the ICUR did not exceed the UAE's willingness-to-pay threshold for any of the input variations, indicating the robustness of the model results.

DSA for SAMe vs. No treatment

**Figure 4:**
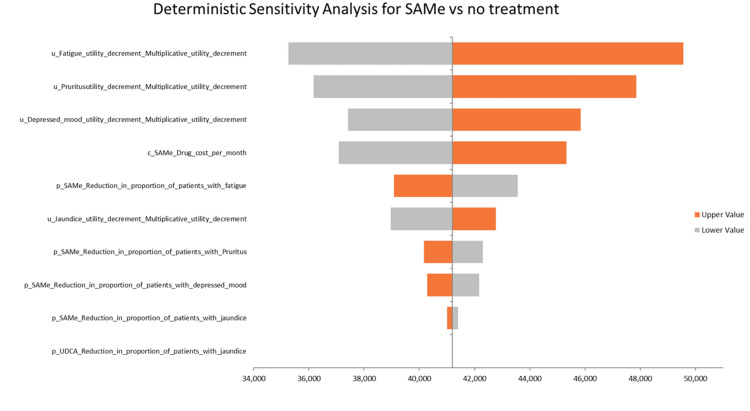
DSA of SAMe vs. no treatment DSA: deterministic sensitivity analysis, SAMe: S-adenosyl-L-methionine

The base case ICUR for SAMe vs. no treatment is 41,202 AED/QALY, which is significantly below the willingness-to-pay threshold of 161,854 AED, indicating a favorable cost-effectiveness profile with a difference of 118,798 AED below the threshold. The most influential parameter is the disutility value of fatigue, as shown in Figure 4, where the ICUR ranges from 35,258 AED to 47,862 AED, corresponding to a percentage change of -14.42% to +16.14% from the base-case ICUR. The second most influential parameter, the disutility value of pruritus, shows an ICUR range of 36,170 AED to 46,000 AED, reflecting a change of -12.22% to +11.64%. The third most impactful parameter, the disutility value of depressed mood, results in an ICUR variation from 37,418 AED to 45,838 AED, representing a change of -9.16% to +11.26%. These results underscore the significant influence of utility decrements related to fatigue, pruritus, and depressed mood on the cost-effectiveness outcomes of SAMe.

DSA for UDCA vs. No treatment

**Figure 5:**
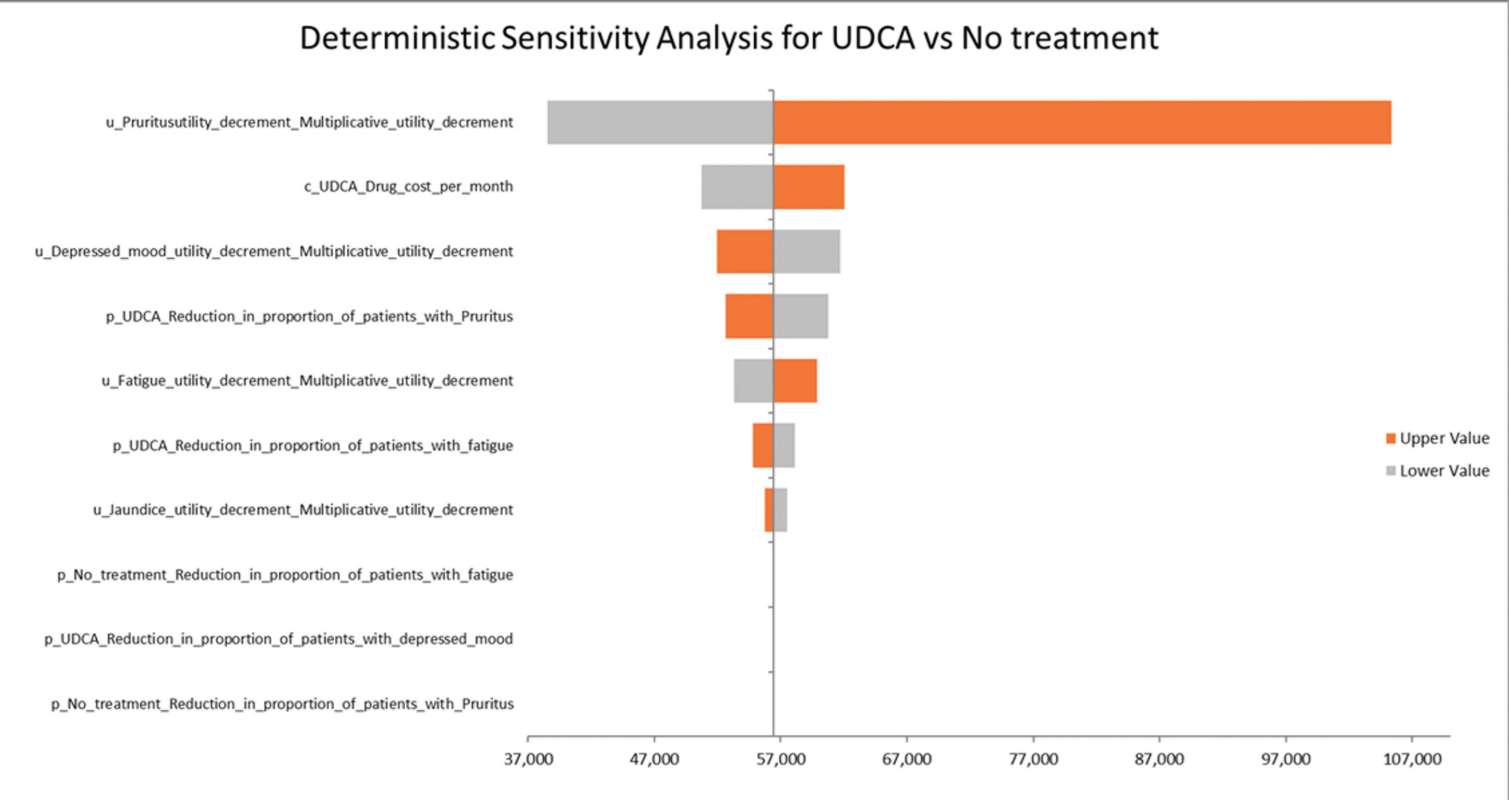
DSA of UDCA vs. no treatment DSA: deterministic sensitivity analysis, UDCA: ursodeoxycholic acid

Based on the base case ICUR of 70,090 AED/QALY for UDCA vs. no treatment, the ICUR is 89,910 AED below the threshold of 161,854 AED, indicating that UDCA is cost-effective under the given threshold. The most influential parameter is the disutility value of pruritus, as shown in Figure 5, where the ICUR fluctuates between approximately 47,850 AED and 130,955 AED, representing a percentage change of -31.73% to +86.76% from the base-case ICUR. The second most influential parameter, the monthly cost of UDCA, has an ICUR range of 63,081 AED to 77,099 AED, corresponding to a percentage change of -10.01% to +10.00%. The third most impactful parameter is the disutility value of depressed mood, with ICER values ranging from 64,531 AED to 76,697 AED, reflecting a change of -7.93% to +9.42%. These findings highlight the critical influence of utility decrements and drug costs on the cost-effectiveness of UDCA vs. no treatment.

Results of the probabilistic sensitivity analysis

**Figure 6:**
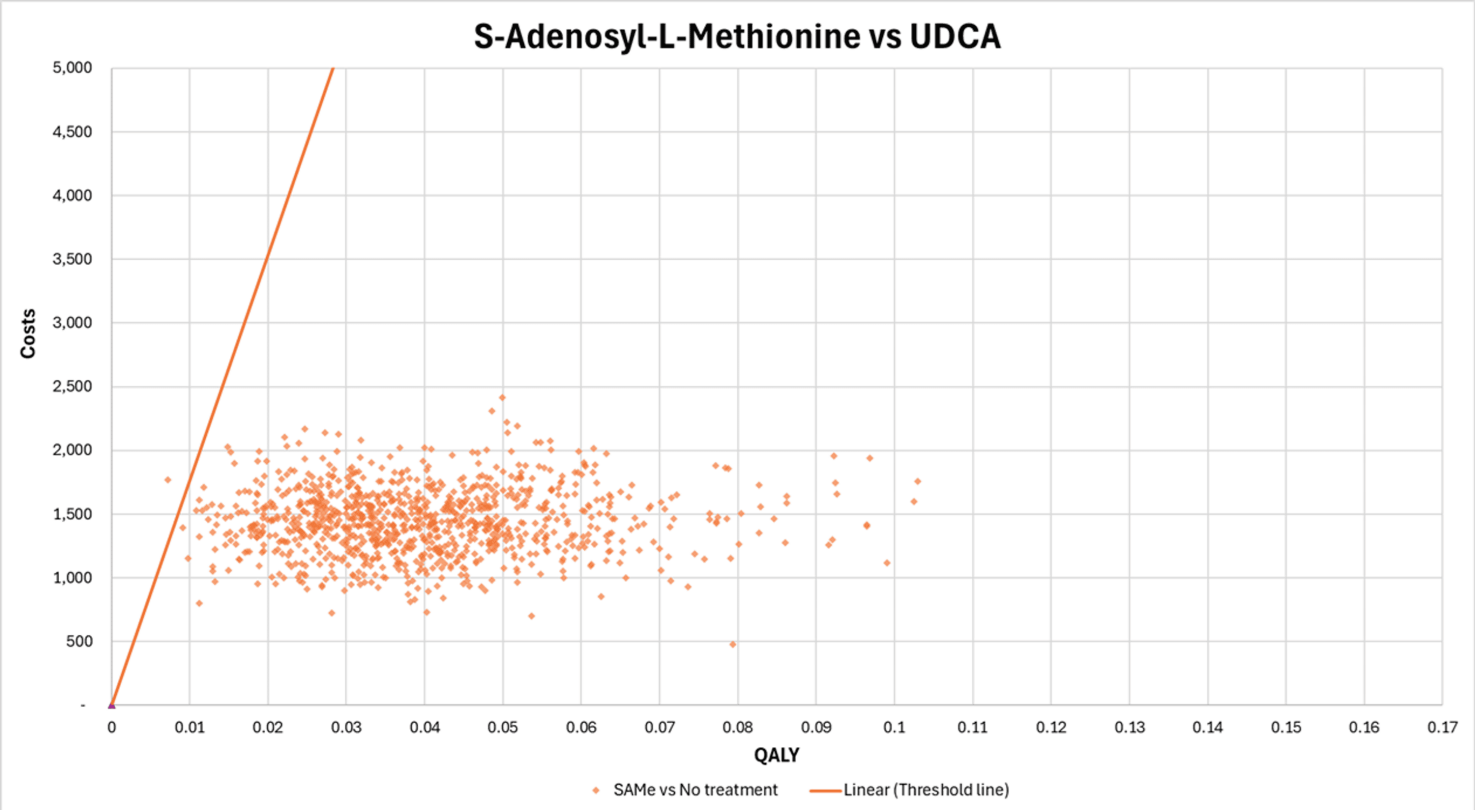
Scatterplot of incremental costs and incremental QALYs on the cost-effectiveness plane (SAMe vs. UDCA) QALYs: quality-adjusted life years, SAMe: S-adenosyl-L-methionine, UDCA: ursodeoxycholic acid

A scatterplot of all 1000 iterations on a cost-effectiveness plane was created for each scenario. The ICUR of SAMe compared to UDCA points are below the threshold line for the majority of the iterations, except six iterations, as shown in Figure 6, indicating that SAMe is cost-effective at the given threshold, providing higher QALYs at an acceptable cost. The consistency of the cost-effectiveness of SAMe suggests that it is a robust option when considering its health benefits.

**Figure 7:**
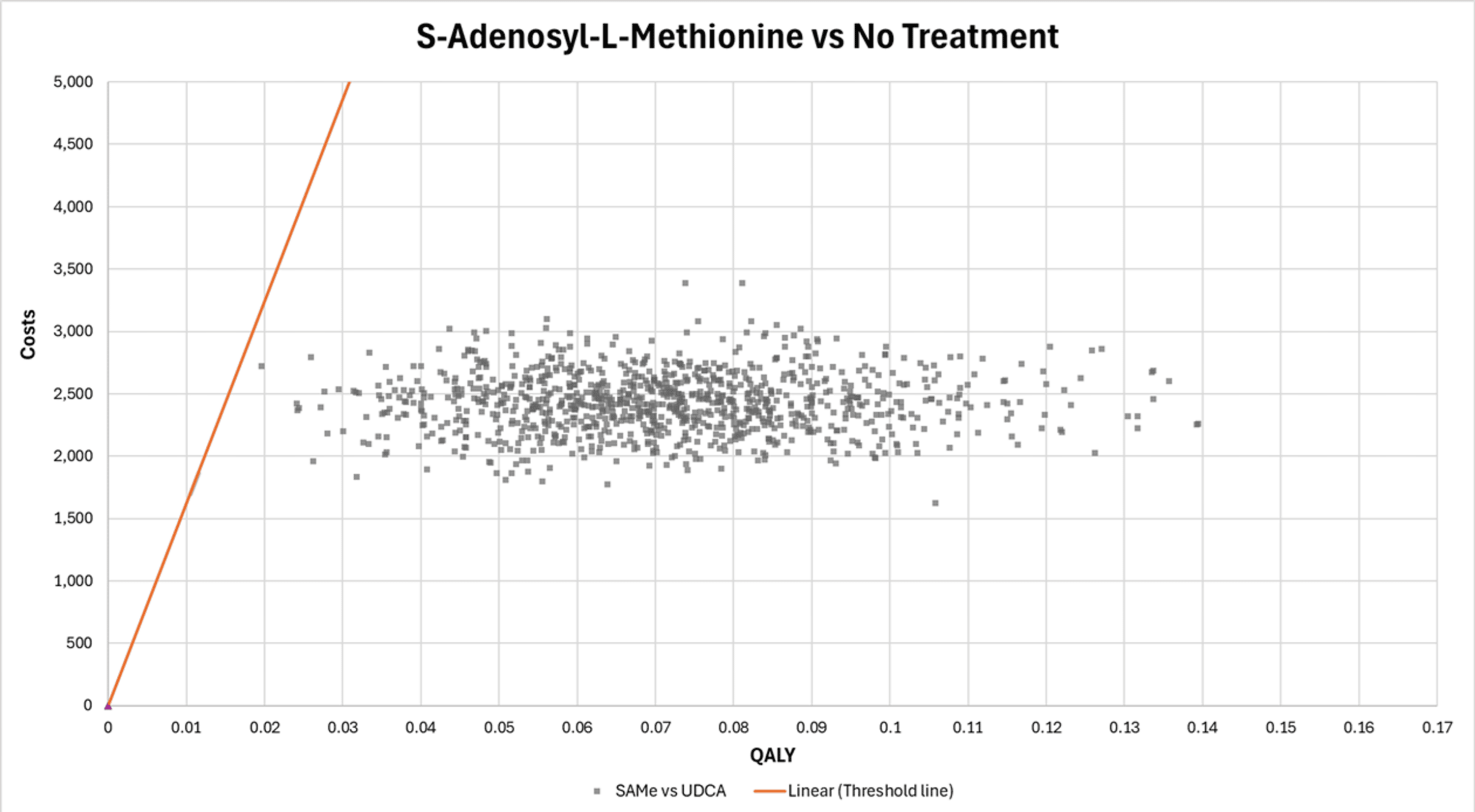
Scatterplot of incremental costs and incremental QALY on the cost-effectiveness plane (SAMe vs. no treatment) QALY: quality-adjusted life year, SAMe: S-adenosyl-L-methionine

A similar scatterplot on the cost-effectiveness plane is shown in Figure 7 for the ICUR of SAMe compared to no treatment. Compared to no treatment, SAMe is shown to be a cost-effective option in all iterations (all iterations lie below the CET line). The consistency of the cost-effectiveness of SAMe suggests that it is a robust option when considering its health benefits.

**Figure 8:**
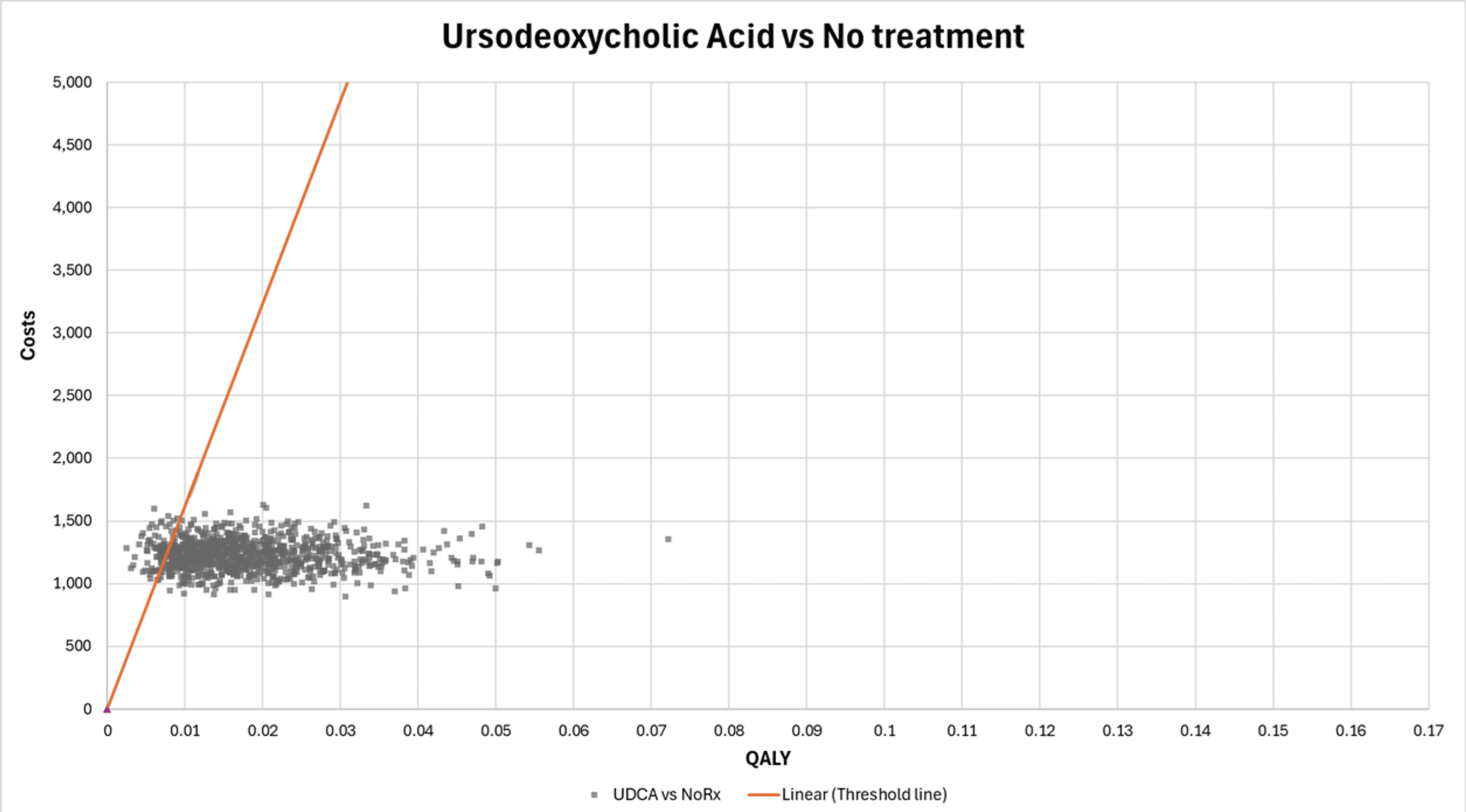
Scatterplot of incremental costs and incremental QALYs on the cost-effectiveness plane (UDCA vs. no treatment) QALYs: quality-adjusted life years, UDCA: ursodeoxycholic acid

A scatterplot on the cost-effectiveness plane is shown in Figure 8 for the ICUR of UDCA compared to no treatment. Compared to no treatment, UDCA is shown to be a cost-effective option in most iterations. However, a substantial proportion of the iterations lie above the CET line. This demonstrates that although UDCA is mostly cost-effective compared to no treatment, there is uncertainty in the results. This uncertainty can favor SAMe over UDCA.

## Discussion

This study assesses the cost-effectiveness and utility of SAMe compared to UDCA for treating IHC using three economic scenarios in the UAE. The head-to-head comparison of SAMe and UDCA indicated that SAMe is cost-effective, despite excluding potential benefits for jaundice and depressed mood due to the absence of evidence regarding UDCA’s effectiveness in treating these conditions. When assessed individually against no treatment, SAMe demonstrated better value for money, as evidenced by a lower ICUR. The robustness of these results was confirmed through both deterministic and probabilistic sensitivity analyses.

SAMe was found to be cost-effective compared to UDCA, with an ICUR of 44,448 AED per QALY. As outlined in the methods, the effectiveness of SAMe in reducing depressed mood and jaundice was excluded from the CUA due to insufficient comparative data for UDCA. Nevertheless, SAMe remained cost-effective. Future investigations should evaluate UDCA’s impact on the depressed mood and jaundice to enhance comparative analyses.

Using no treatment as a baseline, SAMe achieved a lower ICUR (41,202 AED/QALY vs. 70,090 AED/QALY). SAMe's ability to address additional comorbidities, such as jaundice and depressed mood, contributed to its more favorable cost-effectiveness profile. In contrast, UDCA's narrower therapeutic scope, based on the available data, limits its use in comprehensively addressing the IHC-associated comorbidities.

Deterministic and probabilistic sensitivity analyses reflected the robustness of the model results. SAMe’s ICUR consistently remained below the UAE’s willingness-to-pay threshold across all input variations. The model's sensitivity to utility decrements for fatigue and pruritus highlights the importance of accurately capturing patient-reported outcomes in economic evaluations.

As for now, no peer-reviewed CEA comparing SAMe with placebo or UDCA (either directly or indirectly) for ICH in non-pregnant adults has been identified. However, a recent preprint article investigating the cost-effectiveness of SAMe compared with placebo and UDCA compared with placebo in China found that SAMe resulted in an additional 3.49 QALYs at CNY263,417 per 100 patients and an ICER of CNY75,423 compared to placebo (cost-effective at the specified threshold). Additionally, UDCA resulted in an additional 0.87 QALYs at an additional cost of CNY358,197 per 100 patients and an ICER of CNY410,361 compared to placebo (not cost-effective at the specified threshold). A Chinese study found that SAMe dominates UDCA with an ICER of -CNY36,175, indicating both better outcomes and cost savings [14].

The study comes with strong points. The multi-scenario approach provides a comprehensive assessment of SAMe's therapeutic and economic impacts. The favorable ICURs observed across multiple scenarios support the integration of this treatment protocol for IHC in the UAE.

Limitations

This study has several limitations that should be noted. One key constraint lies in the limited availability of clinical trial data, particularly regarding the impact on comorbidities associated with IHC. While SAMe has demonstrated evidence of reducing symptoms such as depressed mood and jaundice, similar data for UDCA are lacking. This presented a methodological challenge: assuming the benefits exist exclusively for SAMe and not for UDCA could have exaggerated SAMe's comparative effectiveness, given the possibility that UDCA may offer some benefit, albeit to a lesser extent. Conversely, excluding the unique benefits of SAMe, where data were unavailable for UDCA, although conservative, likely underestimates SAMe’s overall effectiveness. In this analysis, we adopted a conservative approach for the base case comparison, incorporating only comorbidities for which data were available for both interventions. A more inclusive scenario analysis, comparing each intervention to no treatment, was used to account for the broader spectrum of SAMe’s benefits.

A second limitation pertains to the model’s short time horizon of six months in the CUAs. The absence of long-term follow-up data and consultations with clinical experts informed this decision, as they highlighted the heterogeneity in disease progression, given that IHC frequently coexists with other hepatic or systemic conditions of varying etiologies. This variability precluded a reliable long-term projection of utility outcomes.

Lastly, the model included only direct drug costs, excluding the costs associated with managing IHC-related comorbidities. This omission likely leads to an overestimation of the ICERs, particularly in scenarios where SAMe demonstrates a superior reduction in comorbidity burden. By not accounting for potential cost offsets related to improved symptom control, the model may undervalue SAMe’s economic advantage.

## Conclusions

The study provides valuable insights into the cost-effectiveness of SAMe for treating chronic IHC in the UAE. SAMe proves more cost-effective than UDCA and no treatment, particularly when considering its broader impact on comorbidities. These findings support its integration into treatment protocols for IHC, with the potential to improve patient outcomes and optimize healthcare resource allocation. Further research is needed to address data gaps, especially regarding UDCA's effects on jaundice and depressed mood. Additional clinical trials and real-world evidence will help refine these recommendations and strengthen the decision-making framework.
